# Tumor Derived Mutations of Protein Tyrosine Phosphatase Receptor Type K Affect Its Function and Alter Sensitivity to Chemotherapeutics in Glioma

**DOI:** 10.1371/journal.pone.0062852

**Published:** 2013-05-16

**Authors:** Supreet Agarwal, Maha S. Al-Keilani, Mohammad A. Y. Alqudah, Zita A. Sibenaller, Timothy C. Ryken, Mahfoud Assem

**Affiliations:** 1 Pharmaceutics and Translational Therapeutics, College of Pharmacy, University of Iowa, Iowa City, Iowa, United States of America; 2 Department of Neurosurgery and Radiation Oncology, University of Iowa, Iowa City, Iowa, United States of America; 3 Department of Neurosurgery, Iowa Spine and Brain Institute, Waterloo, Iowa, United States of America; University of Chicago, United States of America

## Abstract

Poor prognosis and resistance to therapy in malignant gliomas is mainly due to the highly dispersive nature of glioma cells. This dispersive characteristic results from genetic alterations in key regulators of cell migration and diffusion. A better understanding of these regulatory signals holds promise to improve overall survival and response to therapy. Using mapping arrays to screen for genomic alterations in gliomas, we recently identified alterations of the protein tyrosine phosphatase receptor type kappa gene (PTPRK) that correlate to patient outcomes. These PTPRK alterations are very relevant to glioma biology as PTPRK can directly sense cell–cell contact and is a dephosphorylation regulator of tyrosine phosphorylation signaling, which is a major driving force behind tumor development and progression. Subsequent sequencing of the full length PTPRK transcripts revealed novel PTPRK gene deletion and missense mutations in numerous glioma biopsies. PTPRK mutations were cloned and expressed in PTPRK-null malignant glioma cells. The effect of these mutations on PTPRK anti-oncogenic function and their association with response to anti-glioma therapeutics, such as temozolomide and tyrosine kinase inhibitors, was subsequently analyzed using *in vitro* cell-based assays. These genetic variations altered PTPRK activity and its post-translational processing. Reconstitution of wild-type PTPRK in malignant glioma cell lines suppressed cell growth and migration by inhibiting EGFR and β-catenin signaling and improved the effect of conventional therapies for glioma. However, PTPRK mutations abrogated tumor suppressive effects of wild-type PTPRK and altered sensitivity of glioma cells to chemotherapy.

## Introduction

Patients with malignant glioma have a poor prognosis due to the widespread infiltration of tumor cells into surrounding healthy brain parenchyma, hyper-vascularization and drug resistance. The majority of glioma patients die within a year of diagnosis because of operative and therapeutic complications mainly resulting from extensive invasion of brain tumor cells [1], [2]. Conventional therapeutic interventions including surgery, radiotherapy and chemotherapy have fallen short of expectations [1], [3]. The shortcomings of conventional therapies call for better understanding of glioma genetics particularly as it relates to key regulatory signals that control cell invasion and migration. Therefore, new insights into regulatory signals playing a vital role in gliomagenesis, progression and invasion are of major interest.

One of the frequently altered regulatory signals in glioma is constitutive protein tyrosine phosphorylation (TP) that drives cell growth and migration [4], [5]. Recent genomic profiling studies have indeed shown overactivation of receptor tyrosine kinase pathways via tyrosine phosphorylation as the most commonly altered phenomena in glioma, with more than 80% of glioma displaying epidermal growth factor receptor (EGFR) constitutive TP and subsequent tyrosine kinase burst [5], [6]. Unchecked TP is instrumental in overactivated cellular processes leading to cell growth, invasion, migration as well as resistance to therapy [4]. Thus, targeting the TP regulatory signals represents a potential therapeutic approach and is important given the fact that the preliminary efficacy results of most clinical trials, targeting tyrosine kinase activity, have fallen short of expectations [3], [7]. Recent studies suggest a key role of protein tyrosine phosphatases (PTPs) mediated dephosphorylation in reducing TP levels in cancer cells [4], [8]. Although functional effects of alterations in PTPs' dephosphorylating activity have been recently reported in human tumors [8], [9], no single PTP study has as yet shown to influence the malignant phenotype and drug response in glioma.

Protein Tyrosine Phosphatase Receptor Type Kappa (PTPRK), one of the 21 known receptor type PTPs, is a transmembrane protein that regulates cell-cell contact. The extracellular region contains a MAM domain, an immunoglobulin like-domain and four fibronectin type III domains, similar to homophilic cell adhesion molecules, essential for cell-cell adhesions [8]. PTPRK mediates highly specific intercellular homophilic interactions suggesting that it can directly sense cell-cell contact and thereby mediate contact inhibition of cell growth [8]. This process is disturbed in many tumors [8], [10]–[13]. Moreover, PTPRK extracellular domain transduces cell-cell contact information across the membrane to the intracellular domains [8]. The intracellular region of PTPRK consists of phosphatase domains with dephosphorylating activity and potential transcriptional modulator function, and thereby regulates tyrosine phosphorylation levels of several targets. Interestingly, the PTPRK locus (6q22–23) is a common region of allelic deletion at chromosome 6 in several cancers [12]–[14]. Indeed, loss of PTPRK activity has been observed in pancreatic cancer, primary CNS lymphoma and melanoma, and is associated with poor survival of cancer patients [8], [10]–[13]. These findings suggest that PTPRK is a potential tumor suppressor, lost in multiple cancers.

In our previous study, we observed frequent and significant alterations of the PTPRK locus in patients with malignant glioma [15]. PTPRK alterations seem relevant to glioma biology as PTPRK is highly expressed in brain. Our data provide first time evidence in support of PTPRK's major role in checking migratory and invasive phenotype of malignant glioma. We identified several inactivating mutations, characterized their functional consequences and effect on pharmacologically relevant PTPRK-dependent molecular pathways along with their prognostic/predictive significance.

## Materials and Methods

### Sequencing

Six LOH-positive glioma biopsies were used (Participants provided their written informed consent. Approved by the University of Iowa institutional review board HawkIRB; IRB#200707727). RNA from these biopsies (5 µg) was reverse transcribed into cDNA using MuMLV retrotranscriptase and oligo dT (Life Technologies, Carlsbad, CA). Fifty nanogram of cDNA was used for further analyses. Primer3 was used to design primers to amplify and sequence full length PTPRK cDNA that were TA cloned (Life Technologies, Carlsbad, CA). We selected several internal primers to sequence full length PTPRK using BigDye Terminator Cycle Sequencing 3730×l (Applied Biosystems, Foster City, CA). The following primers covering different regions of the PTPRK transcripts were used for sequencing: PTPRK-534 (AATGGGTGCATGTTAGTGCTC and GAGCACTAACATGCACCCATT), PTPRK-1791 (CTTCCCTCTGGATTGGTTAGG and CCTAACCAATCCAGAGGGAAG), PTPRK-2770 (TGCATTCACCATGTGAGTCAT and ATGACTCACATGGTGAATGCA), and PTPRK-4380 (TGCACCATCAGATAACCTTCC and GGAAGGTTATCTGATGGTGCA). PCR reactions were performed under the following conditions: 32 cycles of 95°C for 30 sec, 55°C for 30 sec, and 72°C for 3 min. Sequence alignment was done using DNA Baser v2 (Heracle BioSoft, Germany). True variant was confirmed only if identified by both forward and reverse primers.

### Cell culture and transfection

Two different cell lines were used as recently recommended by the cancer research community. U87-MG cell line (ATCC, Manassas, VA) and U251-MG cell line (gift from Dr. Maltese [16]) were maintained in DMEM supplemented with 10% fetal bovine serum, 1% penicillin/streptomycin, and 1% glutamine at 37°C in 5% CO_2_. Wild-type PTPRK and variants were cloned into the expression plasmid pIRES-hrGFP-II (Stratagene, La Jolla, CA) and transfected into glioma cell lines using X-fect transfection reagent (Clontech, Palo Alto, CA). Mock cells were generated by transfecting the empty vector as control.

### Drugs

Three drugs, temozolomide (Sigma-Aldrich, St. Louis, MO), erlotinib (gift from Dr. Simons-Burnett) and gefitinib (Selleckchem, Houston, TX) were used in our studies. They were diluted in 0.25% DMSO for the highest concentrations and serial dilutions were made in the culture media.

### Western blot

Cells were washed twice with ice-cold HBSS, scrapped from the culture plates in 500 µl of RIPA lysis buffer supplemented with 1× protease inhibitor cocktail and 1 mM sodium orthovanadate. Cell lysates were quantified using the bicinchoninic acid assay with bovine serum albumin as a standard. Total homogenates (10–100 µg) were separated on 7.5% polyacrylamide gels and immunoblotted with appropriate primary antibodies (PTPRK, EGFR, β-catenin and GAPDH) and mouse or rabbit peroxidase-coupled secondary antibodies. To analyze PTPRK expression, PTPRK primary antibody recognizing an epitope on the intracellular domain (Ab13325, Abcam, Cambridge, MA) was used along with a PTPRK antibody, recognizing the extracellular domain (gift from Dr. Fisher at the University of Michigan, Ann Arbor, MI). Primary antibodies for total EGFR and β-catenin were purchased from Selleckchem (Houston, TX). Nitrocellulose membranes were developed with the ECL detection system (GE Healthcare, Piscataway, NJ). Band intensities were quantified with Alpha Imager software (Alpha Innotech, San Leandro, CA) using Spot-Denso application.

### Tyrosine phosphatase assay

Tyrosine phosphatase activity was determined using an assay adapted from Xu et al. [17]. In this assay, phosphatases catalyze the hydrolysis of colorless substrate, p-nitrophenyl phosphate (pNPP), to yellow color p-nitrophenol. Briefly, Phosphatase assay buffer (100 µl) containing 15 mM p-nitrophenyl phosphate (NEB, Ipswich, MA), 100 µg/ml bovine serum albumin, 50 mM Tris, pH 7.6, 100 mM NaCl, and 10 mM EDTA was mixed with total cell lysate (0–50 µl) and incubated at 37°C for 40 minutes in 96-well microtiter plates. Reactions were terminated by the addition of 10 N NaOH. Hydrolysis of p-nitrophenyl phosphate was measured spectrophotometrically at 405 nm using a microplate reader (Spectra Max Plus, Molecular Devices, CA). The assay was first validated by measuring phosphatase activity using U87-MG cells transfected with wild-type PTPRK vector [17]. Sodium fluoride at 5 mM final concentration was used to inhibit serine/threonine phosphatases activity that may interfere with measurements. Additionally, specificity of the assay for tyrosine phosphatases was confirmed with orthovanadate, a tyrosine phosphatase inhibitor, which inhibited phosphatase activity of U87-MG mock cells and PTPRK transfected cells to similar level.

### Proliferation and migration assay

A MTT colorimetric assay was used to measure cell proliferation (Sigma-Aldrich, St. Louis, MO). The wound healing assay was performed to determine differences in cell migration rates of U87-MG and U251-MG cells transfected with either wild-type PTPRK or PTPRK variants. Individual wounds were made with a 10 µl pipette tip. Cell images were taken using a Jenco microscope (Jenco, Portland, OR) equipped with a Mightex CCD camera at 0 and 24 h. Image analysis was done using TScratch software (CSElab, ETH Zurich, CH) and changes in wound areas per time were measured.

### Cell invasion assay

U87-MG and U251-MG cells were plated in 6-well plates and transfected with wild-type PTPRK and its variants for 48 h. The cells were trypsinized, centrifuged and resuspended into serum-free medium at 1×10^5^ cells/ml. 500 µl of transfected cells were seeded into the matrigel-coated and rehydrated filter inserts of 24-well transwell chambers with 8 µm pores (BD Biosciences, Bedford, MA). Bottom chambers were filled with 750 µl of DMEM containing 10% FBS. After 24 h incubation at 37°C, filter inserts were removed, the medium was decanted, and the inserts were washed twice with PBS. Non-invading cells on the upper surface of the membrane were scrubbed off with a cotton swab. The invasive cells on the lower surface of the membrane were fixed with 100% ethanol for 5 minutes and stained with 0.1% crystal violet solution in 20% ethanol for 30 minutes. Stained cells were washed twice with PBS and counted in five random fields under a light microscope.

### Cytotoxicity assay

For cytotoxicity assay, U87-MG and U251-MG cells were first transfected with wild-type PTPRK and its variants for 48 h in a six well plate. The cells were trypsinized with 0.05% trypsin and plated at a density of 4000 cells/well in a 96 well plate. After 24 h incubation at 37°C, cells were treated with temozolomide (100 µM), eroltinib (10 µM) or their combination for 72 h with each variant having six replicates per treatment condition. Similar treatment was performed with temozolomide (100 µM), gefitinib (10 µM) or their combination in a separate 96 well plate. The growth inhibition effect of these drugs was analyzed using MTT cytotoxicity assay. Briefly, after 72 h of drug treatment, 10 µl of MTT solution (final concentration 0.5 mg/ml, Sigma) was added to each well and the plates were incubated for 4 h at 37°C. The resultant formazan product was dissolved in DMSO and absorbance was measured at 540 nm with background absorbance at 690 nm using a microplate spectrophotometer (Spectra Max Plus, Molecular Devices, CA). The results represent the mean ± SE values, from two independent experiment, each with six replicates per variant per treatment condition, expressed as a percentage of viable cells relative to untreated mock cells (Mock+DMSO).

### Statistics

Statistical significance was determined using non-parametric ANOVA (SAS 9.3 software) with significance level of p-value<0.05. Multiple group comparison was done using non-parametric analog to the Tukey-Kramer method. Simple effects test was done as a follow-up test to interpret interactions between drug treatment and PTPRK variants for demonstrating PTPRK induced chemotherapeutic response. Bonferonni correction was used to adjust the critical alpha level and thus P-value<0.01 was considered for statistical significance.

## Results

We recently characterized several copy number alterations and losses of heterozygosity (LOH) in a comprehensive population of malignant glioma tumor specimens [15]. We discovered that alterations of the PTPRK gene were among the most significant abnormalities. Moreover, overall survival analysis identified PTPRK as a tumor suppressor candidate with prognostic, therapeutic and clinical predictive values [15]. Interestingly, previous studies have also suggested association between altered PTPRK genomic status and poor survival in other cancers [8], [12], [13]. Despite a potential key role of PTPRK in glioma biology, functional data related to PTPRK alterations in gliomagenesis are lacking. Thus, we identified PTPRK as a candidate for further validation.

### Sequence analysis of the PTPRK coding region revealed variants with altered functional activity

SNP arrays do not allow identification of particular coding variation because they exclusively use intronic probe sets (Mapping array SNP 6.0, Affymetrix, Santa Clara, CA). They only provide an indirect estimation of genetic alterations in the coding regions. In our recent study, LOH at the PTPRK locus was observed in 23% of glioma patients [15]. However, microarray screening is often associated with false positive results and requires further confirmation. Therefore, we decided to confirm and validate our data using direct sequencing of the PTPRK coding region. Six LOH positive specimens were randomly selected for confirmation studies. Tumor cDNAs were PCR amplified and sequenced. As shown in Table 1, sequence alignment detected two non-synonymous mutations (A3938G (T1184A) and A4064G (S1226G)) in the phosphatase domain-2 and three synonymous in the fibronectin-III domain (Table 1 and Fig. S1). To predict whether these variations are likely to alter the protein's function, we used the SIFT web application (http://sift.jcvi.org/). Evaluation of PTPRK mutations by SIFT showed that T1184A is a ‘damaging’ mutation (SIFT score = 0) whereas S1226G is ‘tolerated’ (SIFT score = 1). These non-synonymous mutations in the phosphatase domain-2 induce switch from polar to non-polar amino acid, and thereby may modify PTPRK function. Moreover, we found a deletion of exons 15 to 22 which encode for a region in the tyrosine phosphatase domain-1. This deletion is bound to alter PTPRK function because a part of the phosphatase domain-1 is missing (∼200 amino-acids). Overall, these alterations could have strong functional implications, possibly resulting in conformational changes of the phosphatase domain or folding of the PTPRK protein that in turn can affect availability of the active site and thereby render the protein less- or non-functional. Biological consequences, functionality and predictive significance of these PTPRK variants were subsequently determined in U87-MG and U251-MG cell lines. Throughout the text, wild-type PTPRK is designated as PTPRK-wt, the variant harboring the mutation A3938G as PTPRK-mut and the variant harboring the exons 15–22 deletion as PTPRK-del.

**Table 1:** Summary of genetic alterations in a subset of glioma specimens.

Nucleotide change	Amino acid change	Functional domain	SIFT prediction	Nomenclature
T1480C	G371G	FN_III	Tolerated	-
T2371C	Y668Y	FN_III	Tolerated	-
C2389A	L674L	FN_III	Tolerated	-
A3938G	T1184A	Phosphatase	**Damaging**	**PTPRK-mut**
A4064G	S1226G	Phosphatase	Tolerated	-
Exons 15–22 (Deletion)	-	Phosphatase	**Damaging**	**PTPRK-del**

The reference sequence NM_001135648.1 was used for annotation of the nucleotide and amino acid changes. Functional consequences of the mutations were evaluated using SIFT program.

### PTPRK variants have lower phosphatase activity

**Figure 1:**
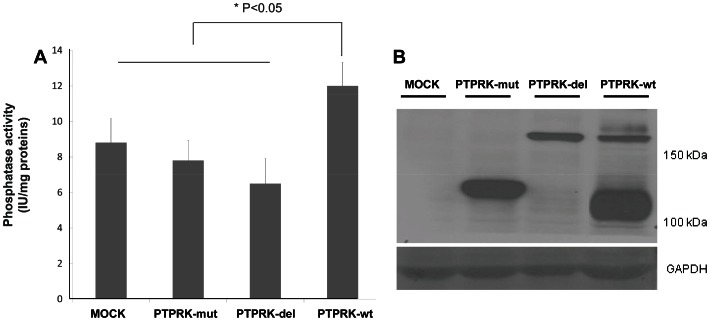
Mutation events in malignant glioma alter PTPRK phosphatase activity and post-translational processing. (A) Wild type PTPRK (PTPRK-wt) and its variants were transiently re-expressed in U87-MG cells. Effect of PTPRK mutations on phosphatase activity is shown. Values are mean ± standard error of 5 assays in each group. Multiple comparisons were performed to determine activity differences between the variants. P<0.05 was considered significant for ANOVA analysis. (B) Western blot of lysates from U87-MG mock cells and cells expressing wild type PTPRK or indicated PTPRK mutations. Immunoblotting was performed using an antibody against the PTPRK extracellular domain, recognizing full length PTPRK (180-kDa) and processed extracellular fragment of size 120-kDa. Immunoblot was reprobed for GAPDH as a loading control.

To study the functional impact of identified genetic variations, we cloned wild-type PTPRK and its variants into an expression vector and performed transient re-expression studies in U87-MG cells. We selected U87-MG cell line because we have shown that it does not express functional PTPRK and therefore is an appropriate *in-vitro* model for analyzing consequences of PTPRK expression. We did not use U251 cells because we have shown that they express moderate amount of endogenous PTPRK which may complicate interpretation of the assay results (in case of dominant negative effect). Moreover, we opted for transient transfection because we were not able to create stably transfected clones as cells die or lose PTPRK expression by 9–12 days after transfection. We examined the effect of these PTPRK mutations on enzymatic activity by p-nitrophenyl phosphatase (pNPP) assay. Difference between variants and mock cells was not significant. Both PTPRK-mut and PTPRK-del showed significant reduction in activity compared to PTPRK-wt (Fig. 1A). PTPRK-mut and PTPRK-del are 35% and 46% less enzymatically active than PTPRK-wt (p = 0.0387). These differences in PTPRK activity could possibly alter growth suppressive and anti-migratory effects among the PTPRK variants.

### PTPRK mutations lead to differential post-translational patterns in glioma cells

Most trans-membrane receptor proteins, including receptor type PTPs, are post-translationally processed into multiple isoforms by proteases such as metalloproteases, furin, α- and γ-secretases [18]–[21]. Initial analysis of PTPRK protein levels in human glioma biopsies confirmed that PTPRK protein is proteolytically processed into several fragments (Fig. S2). Full length PTPRK (180-kDa) was not detectable in a majority of the analyzed glioma biopsies. However, high levels of low molecular weight isoforms, corresponding to extracellular (120-kDa) and intracellular PTPRK fragments (∼75-kDa and 64-kDa), were detected. Additional bands of cleaved PTPRK were seen which are yet to be characterized. In addition, western blot analysis of total cell lysates from U87-MG cells, transfected with wild-type PTPRK or the two variants, showed differential post-translational PTPRK processing (Fig. 1B). PTPRK-wt showed two bands; a) a 180-kDa band corresponding to the full length PTPRK protein and b) the lower 120-kDa band corresponding to the extracellular region of processed PTPRK. PTPRK-mut is 100% processed as indicated by a single fragment of size 120-kDa, whereas, PTPRK-del is not processed as a single band of around 180-kDa was observed. Our results showed that mutation events modify PTPRK processing, possibly by changing susceptibility to proteases such as furin, α- and γ-secretases. These modifications could alter PTPRK regulated cell proliferation and migration.

### Mutations alter growth suppressive, anti-migratory and anti-invasive properties of PTPRK in glioma

**Figure 2:**
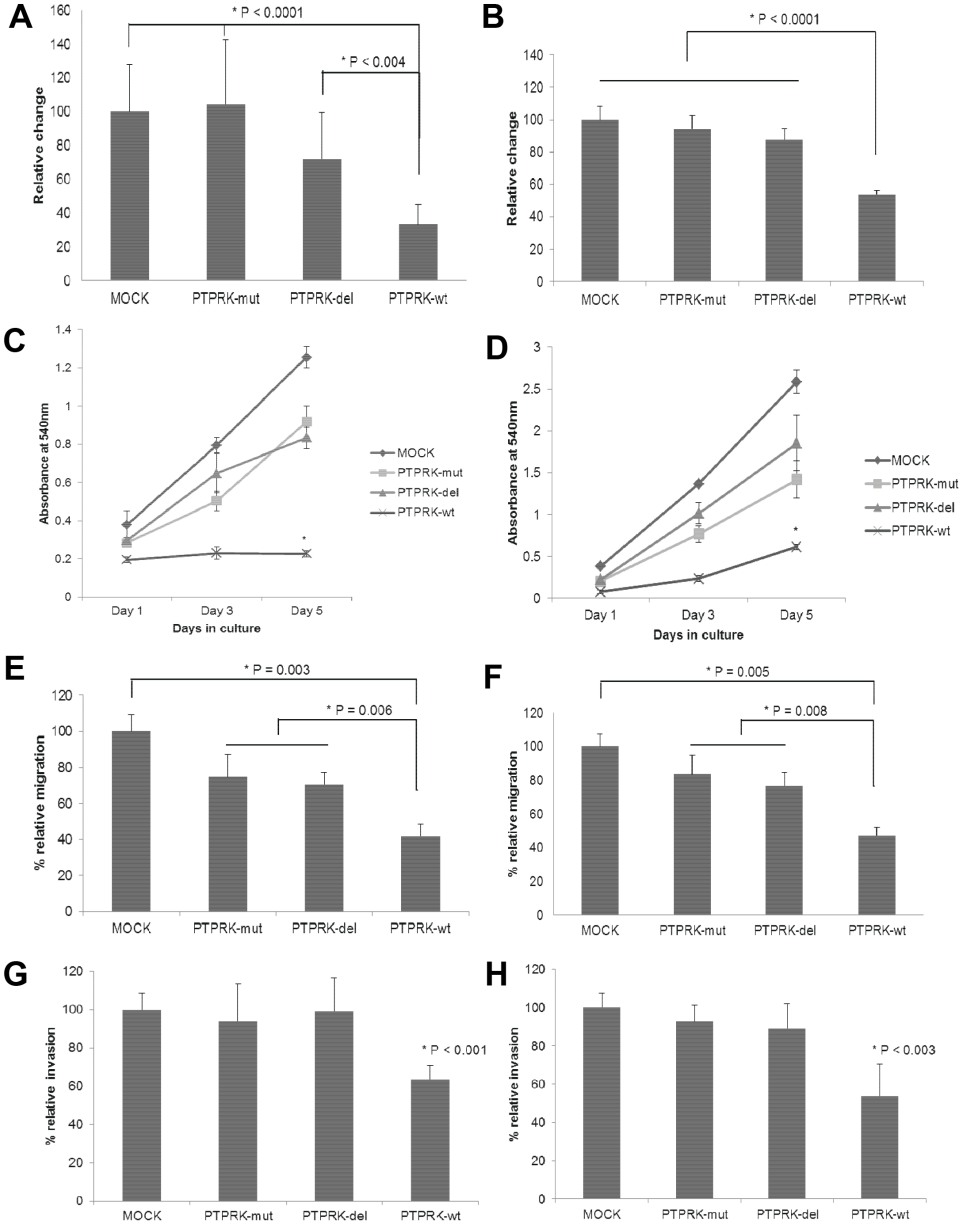
PTPRK variants alter suppressive effect of PTPRK-wt on growth, migration and invasion of glioma cells. Quantitative assessment of growth suppressive properties of PTPRK-wt and effect of variants was determined by MTT colorimetric assay in (A) U87-MG and (B) U251-MG cells. The data represent mean ± standard error of six independent experiments performed in triplicate. P<0.05 was considered significant. Growth of U87-MG and U251-MG cells, expressing PTPRK-wt or PTPRK mutants, is plotted in (C) and (D). Cell growth was assessed by crystal violet staining at indicated time points and quantified by measuring absorbance at 540 nm of methanol-solubilized cell stain. The results are plotted as the average growth (A_540 nm_) ± standard error and * indicates statistical significance of PTPRK-wt in comparison to mock or PTPRK variants (P<0.05). Migration analysis using wound healing assay is shown in (E) and (F) for U87 and U251 cells, respectively. Changes in wound area per time were measured in glioma cells transfected with empty (N = 15), mutants (N = 15) or PTPRK-wt (N = 15). Invasion of (G) U87-MG and (H) U251-MG cells, expressing PTPRK or its mutant forms, was assessed by matrigel invasion assay. Histogram shows average count of invaded cells for each PTPRK clone as a percentage of invading mock cells. Multiple comparisons were performed to determine significant differences between the variants and P<0.05 (*) was considered significant for ANOVA analysis.

We hypothesized that alteration in PTPRK function contributes to the loss of contact inhibition of cell growth, facilitating proliferation and migration of tumor cells throughout the brain parenchyma. The functional consequences of discovered genetic alterations were thus assessed by examining their role in glioma cell growth, migration and invasion which are the key biological features of malignant gliomas. We performed re-expression experiments by transfecting the PTPRK variants into U87-MG and U251-MG cells. PTPRK-wt expressing cells showed significant reduction in cell number compared to PTPRK variants as well as compared to mock cells at 72 h after transfection (Fig. 2A, Fig. 2B and Figs. S3A and S4A). Quantitative analysis of PTPRK-wt growth suppressing property showed a significant (60–70%) decrease in cell proliferation compared to the PTPRK variants in U87-MG cells (Fig. 2A, P<0.0001; PTPRK-wt vs. PTPRK-mut, P<0.004; PTPRK-wt vs. PTPRK-del). Similar level of differences in proliferation among PTPRK isoforms were also observed in U251-MG cells (Fig. 2B, P<0.0001; PTPRK-wt vs. variants). Further, PTPRK-wt expressing cells had significantly lower growth than mock cells (Figs. 2A and 2B, P<0.0001; PTPRK-wt vs. mock). However, PTPRK-mut and PTPRK-del had no influence on proliferation compared to mock cells (P = 0.215). These findings were further validated by comparing growth curves of cells expressing either the PTPRK-wt or its variant forms (Figs. 2C and 2D).

Malignant glioma cells expressing PTPRK-wt proliferated at a significantly reduced rate, resulting in 80% difference in growth compared to mock cells (Figs. 2C and 2D, P = 0.0002). Additionally, PTPRK-wt expressing U87-MG and U251-MG cells had growth rate 60–70% less than that of PTPRK variant expressing cells (Figs. 2C and 2D, P<0.001).

Further, we determined consequences of ectopic expression of PTPRK and its variants on diffusive characteristic of glioma cells with *in vitro* wound healing assay. Reconstitution of wild-type PTPRK significantly (P = 0.003) reduced cell migration of PTPRK-null U87-MG cells by 60% compared to mock cells (Fig. 2E). Parallel to the MTT proliferation assay results, the anti-migratory effect of PTPRK was abrogated for PTPRK-mut and PTPRK-del (Fig. 2E, P = 0.006). The effect of PTPRK variants on migratory nature of U87-MG cells was not statistically significant. (P = 0.076 and P = 0.052, respectively). Moreover, the rate of cell migration was not different between the PTPRK variants (P = 0.4). PTPRK induced inhibition of glioma migration and effect of its mutations were confirmed in U251-MG cell line (Fig. 2F and Figs. S3B and S4B).

We next evaluated the role of PTPRK and its variants in glioma invasion using matrigel invasion chambers. As shown in Fig. 2G, PTPRK-wt expression significantly reduced glioma cell invasiveness by 40% (P<0.001). On the contrary, compared to wild-type PTPRK, expression of PTPRK variants failed to suppress invasive characteristics of U87-MG cells (Fig. 2G and Fig. S3C). These findings were further confirmed in U251-MG cells. PTPRK-wt expressing cells showed 45% less invasion (P<0.003) and the mutations abrogated this anti-invasive effect of PTPRK (Fig. 2H and Fig. S4C). Overall, the data indicate that mutations and deletions of the PTPRK gene alter its suppressive effect on the invasive phenotypes of malignant glioma.

### PTPRK expression sensitizes glioma cells to anti-glioma therapeutics

Since we observed a major role of PTPRK in checking glioma aggressiveness (proliferation, diffusion and invasion), and as malignant gliomas are characterized by a remarkably high chemo-resistance, we analyzed whether PTPRK status modulates the chemosensitivity of malignant glioma cells. We selected temozolomide (current standard of care) and two tyrosine kinase inhibitors (TKis), erlotinib and gefitinib. These TKis are currently being used in several glioma clinical trials as agents for reducing excessive TP that is frequently observed in glioma [4], [5], [22], [23]. Our hypothesis is that tumors with normal PTPRK will respond better to TKi-based therapies whereas tumors with altered PTPRK will be refractory. The synergistic effect of TK inhibition and reduction of tyrosine phosphorylation levels by active PTPRK could increase responsiveness to the TK inhibitors.

**Figure 3:**
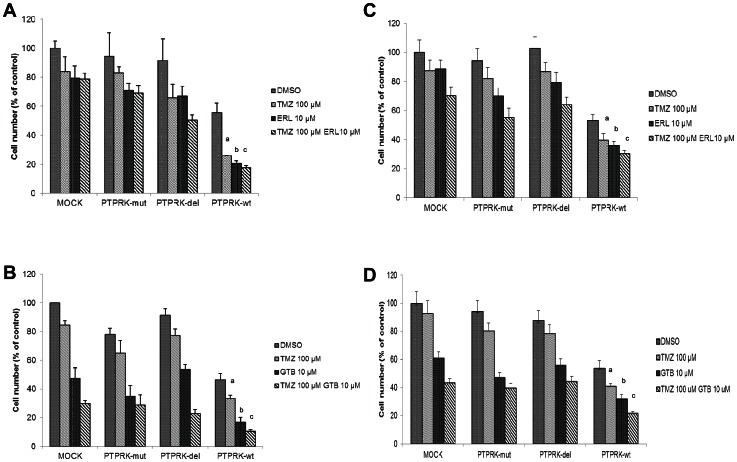
PTPRK variants alter response to anti-glioma agents. U87-MG (A) and U251-MG (C) cells expressing PTPRK-wt or variants were treated with temozolomide (100 µM), erlotinib (10 µM) or their combination. Additionally, U87-MG (B) and U251-MG (D) were treated with temozolomide (100 µM), gefitinib (10 µM) or in combination. MTT assay was performed to assess viability. Data are represented as means ± standard error of six determinants per condition from two independent experiments. Significant differences between PTPRK-wt vs. mock and PTPRK-wt vs. variants is indicated by ‘a’ for temozolomide (TMZ) treatment; ‘b’ for treatment with either erlotinib (ERL) or gefitinib (GTB), and ‘c’ for combination treatment with temozolomide and tyrosine kinase inhibitor (TMZ+ERL, TMZ+GTB). P<0.01 was considered significant.

As expected, using MTT cytotoxicity assay, we observed increased sensitivity of malignant glioma cells to anticancer drugs following expression of functionally active wild-type PTPRK and little or no significant change in sensitivity with abnormal PTPRK, compared to mock cells. Specifically, re-introduction of PTPRK-wt in U87-MG cells resulted in about 71% decrease in cell number upon treatment with temozolomide relative to treated mock cells (P<0.001) (Figs. 3A and 3B). Whereas, PTPRK-mut and PTPRK-del expression reduced cellular growth only by 15–20% indicating moderate improvement (PTPRK-mut, P = 0.1; PTPRK-del, P = 0.08). Similarly, treatment with erlotinib or gefitinib showed better response in PTPRK-wt expressing cells than PTPRK-null or PTPRK mutant-expressors. PTPRK-wt enhanced cytotoxic effects of erlotinib and gefitinib by 75% and 65%, respectively, compared to treated mock cells (erlotinib, P<0.001; gefitinib, P<0.0001) (Figs. 3A and 3B). Furthermore, we evaluated benefits of combination therapy of temozolomide with erlotinib or gefitinib. The combination therapy increased cell death even in PTPRK-null mock cells (Figs. 3A and 3B). In comparison to PTPRK-null mock cells, reconstitution of PTPRK further increased sensitivity by 54% to treatment with combination of temozolomide and gefitinib/erlotinib (Figs. 3A and 3B). However, the two variants altered the singular and combined effects of these drugs. Differences between mock and wild-type PTPRK-expressing cells in their response to each drug treatment condition (temozolomide/erlotinib/gefitinib alone or indicated combinations) were statistically significant (simple effect tests; P<0.01). PTPRK induced chemosensitivity of glioma cells was further confirmed in U251-MG cell line and were consistent with findings in U87-MG cells (Figs. 3C and 3D). Since the observed drug effects could merely be secondary to the anti-proliferative effects of PTPRK, we plotted our data after controlling for effect on cell growth due to ectopic expression of PTPRK and variants (Fig. S5). These results clearly indicate that PTPRK-wt significantly improves existing clinical response to conventionally used anti-cancer agents and tyrosine kinase inhibitors.

### PTPRK alterations modify expression of the PTPRK targets EGFR and β-catenin

**Figure 4:**
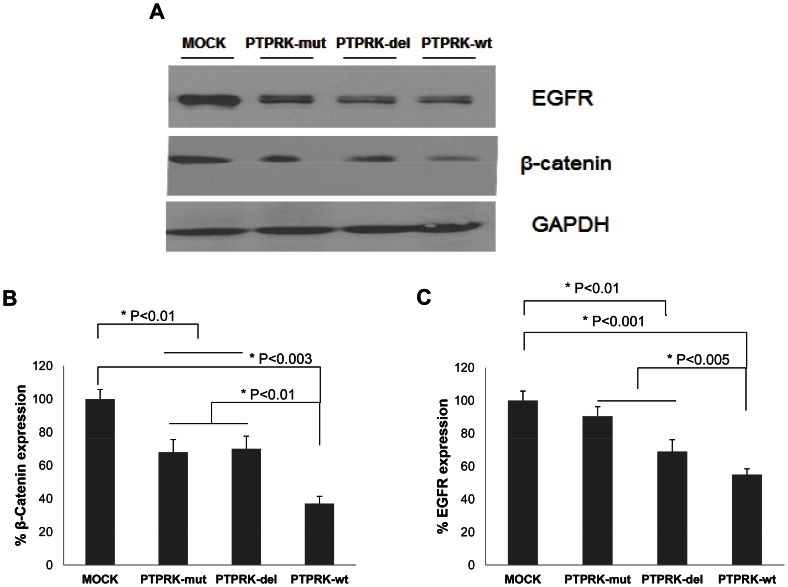
Reconstitution of PTPRK in U87-MG cells altered the expression of β-catenin and EGFR. U87-MG cells transfected with PTPRK-wt, PTPRK-mut and PTPRK-del were assessed for the levels of β-catenin (A and B) and EGFR (A and C) by immunoblot. β-catenin (B) and EGFR (C) protein levels quantified by densitometry are shown as a percentage relative to the control group.

PTPRK is involved in cell-cell contact-mediated signaling pathways both directly through intercellular homophilic binding and indirectly through its dephosphorylating activity [24]. Based on previous studies, we hypothesized that PTPRK may regulate growth, invasion and migratory phenotype of glioma cells through tyrosine dephosphorylation of β-catenin and receptor tyrosine kinases such as EGFR. Interestingly, our results indicate that reconstitution of PTPRK-wt in PTPRK-null U87 cells negatively regulates β-catenin and EGFR expression. PTPRK-wt protein significantly reduced total EGFR and β-catenin protein levels (Fig. 4, 40% and 60% vs. mock, respectively, P<0.001). PTPRK mutants though reduced EGFR and β-catenin levels (PTPRK-mut: 13% (P = 0.1) and 35% (P<0.01), PTPRK-del: 33% (P<0.01) and 35% (P<0.01), respectively) compared to mock cells but clearly not as much as PTPRK-wt (P<0.05). Differential regulation of PTPRK targets as a consequence of deletions/non-synonymous mutations and altered proteolytic processing correlate with observed effects of these variants on glioma cell proliferation and migration as shown in Fig. 2. These results suggest that PTPRK negatively regulate EGFR and β-catenin expression likely resulting from a decrease in tyrosine phosphorylation of EGFR and β-catenin which has been previously reported in other cancer types [17], [25]–[27].

## Discussion

Our recent SNP array screening of glioma biopsies implicated PTPRK as an independent prognostic factor in malignant glioma [15]. We observed that the PTPRK locus undergoes allelic loss in glioma which may contribute to the cancer phenotype. Interestingly, glioma patients with deleted or inactivated PTPRK have poor overall survival compared to those with normal PTPRK locus [15]. Further, inactivation of PTPRK has been recently observed in several tumors [12], [13], indicating and independently confirming its tumor suppressive role. This current extension of our investigations into the role of PTPRK in malignant glioma illustrates its biological relevance as a negative regulator of glioma proliferation, migration and invasion. Moreover, the physiological role of PTPRK as a putative tumor suppressor and its capacity to influence the malignant glioma phenotype has not been studied to date although PTPRK is highly expressed in the brain.

We identified several synonymous, non-synonymous mutations and deletion in the PTPRK gene, and focused on the most ‘damaging’ phenotype. These genetic alterations influenced PTPRK dephosphorylation and adhesion properties and are responsible for major alterations in PTPRK mediated suppression of glioma cell migration, diffusion and invasion. Moreover, we discovered that the PTPRK genetic status can predict *in vitro* response to anti-glioma therapies such as temozolomide and several tyrosine kinase inhibitors.

Similar to other cell surface receptor proteins [18], [19], [28], we observed that PTPRK protein is sequentially processed in glioma by three cleavage events involving 1) a furin-like protease, yielding a PTPRK P-subunit fragment non-covalently attached to the extracellular domains, PTPRK E-subunit; 2) an α-secretase, yielding a PΔE subunit (P-subunit without most of the extracellular region); and, 3) further processing by a γ-secretase ultimately generates a PTPRK-intracellular domain (ICD) fragment (Fig. S2). These post-translational changes could alter PTPRK function depending upon its subcellular localization and accessibility to substrates. A limited number of receptor type PTPs' studies have shown some evidence of the intracellular domain (ICD) also having a potential transcriptional modulating activity upon nuclear translocation [18], [19]. Based on these limited studies, it could be speculated that the processed PTPRK fragment in the cytoplasm, once freed from the membrane, translocates to the nucleus and may contribute to cancer phenotype via undefined mechanisms. Little is known, however, as to the specific functional relevance of such proteolytic processing in human gliomas.

Previous studies indicate that PTPRK regulates cell-adhesion through tyrosine dephosphorylation of β-catenin leading to its localization at adherens junctions, and thereby inhibiting transcriptional activation of its downstream targets [11], [25], [29]. β-catenin signaling plays an important function in cell migration and contributes to tumor development [25]–[27], [30]. Tyrosine phosphorylation of β-catenin (pY-654) results in its disruption from cadherin-catenin complexes and subsequent increase in the cytoplasmic and nuclear pool of free β-Catenin [25], [27], [29]. The free β-catenin drives gene expression of key cell cycle regulator gene cyclin D1 and c-Myc, ultimately contributing to malignant transformation of normal cells [25], [31]. Further, PTPRK plays a critical role in regulating EGFR signaling via tyrosine dephosphorylation of EGFR [17]. Previous reports show that PTPRK plays an antagonistic role in growth factor signaling by dephosphorylation at a tyrosine residue of the EGFR tyrosine kinase domain (at EGFR pY-1173) and thus modulates its downstream signal transduction [17], [32]. EGFR and β-catenin signaling are the only two major pathways that have been studied as potential PTPRK targets in few published cancer studies. Based on the facts that 1) PTPs are believed to have tissue-specific functions, 2) EGFR and β-catenin signaling are among the most frequently deregulated oncogenic pathways in glioma and 3) PTPRK is highly expressed in brain, we hypothesized that PTPRK exerts anti-glioma effect by inhibiting EGFR and β-catenin oncogenic signaling.

Interestingly, PTPRK-wt expression reduced EGFR and β-catenin protein levels in glioma. This possibly suggests that apart from catalyzing dephosphorylation of EGFR and β-catenin, PTPRK also regulates their protein expression in malignant glioma by interacting directly with transcription or by negative feedback-loop after dephosphorylation of EGFR and β-catenin. Alternatively, PTPRK can possibly sense cell-cell contact by trans- homophilic interactions and mediate contact inhibition in cells dependent on EGFR and β-catenin activity for proliferation and migration. Thus, re-expression of PTPRK and consequent intercellular homophilic interactions in glioma cells might induce contact inhibition of growth and thereby send downstream signals to reduce EGFR and β-catenin levels. Further investigations are required to clearly delineate whether PTPRK plays a dual role as a negative transcriptional regulator and as a tyrosine phosphatase of EGFR and β-catenin signaling. Moreover, PTPRK mutations altered the inhibitory effect of wild type PTPRK on EGFR and β-catenin signaling pathways which correlate with observed alterations in PTPRK mediated suppressive effects on growth and migration of glioma cells. Indeed, beside the regulation of EGFR and β-catenin function, other not known signaling processes could be involved, leading to inhibition of phenotypic hallmarks of glioma. However, EGFR and β-catenin are the only known substrates of PTPRK so far. Screening of PTPRK substrates using commercially available “tyrosine phosphatase substrate sets” and subsequent validation of identified substrates in relation to their relevance in glioma biology is required for better understanding of PTPRK regulated molecular events.

Ectopic expression of PTPRK-wt in glioma cells showed significant reduction in the proliferation, migration and invasion rates compared to PTPRK variants possibly via negative regulation of EGFR and β-catenin signaling. Our results suggest that tumor derived mutations of the PTPRK gene significantly alter PTPRK functionality, and thereby the PTPRK regulated signaling cascade by two ways: 1) altering its phosphatase activity; and, 2) altering post-translational processing of PTPRK. Genetic alterations (PTPRK-mut and PTPRK-del) resulted in significantly lower phosphatase activity compared to PTPRK-wt. PTPRK-mut alteration is exclusively in the phosphatase domain-2 which is catalytically inactive but regulates enzymatic activity of the phosphatase domain-1. The deletion in PTPRK-del encompasses a region in phosphatase domain-1 prior to the conserved catalytic motif critical for phosphatase activity. This may explain the partial reduction in enzymatic activity without a complete loss. Furthermore, we demonstrated that PTPRK mutations (PTPRK-mut) result in 100% post-translational processing of full length PTPRK protein potentially leading to several PTPRK isoforms (PΔE and intracellular domain) whereas PTPRK-del showed no post-translational processing. The deletion might have resulted in conformational changes in the PTPRK protein leading to resistance to proteases. As hypothesized, these alterations in PTPRK phosphatase activity and post-translational modification altered the suppressive effects of PTPRK on glioma growth, migration and invasive phenotypes. Interestingly, though not statistically significant, PTPRK-mut and PTPRK-del showed decrease in relative migratory activity which could be explained based on partial but not complete loss of phosphatase activity and post-translational processing of these variants. Specifically, PTPRK-del showed no proteolytic processing thus could be localized exclusively on the cell membrane. Therefore, PTPRK-del can induce contact mediated inhibition of glial cell migration via homophilic interactions. However, weak homophilic interactions due to mutations, together with reduced phosphatase activity can subsequently result in weak or altered transduction of downstream signals in response to migrating glial cells. PTPRK-mut, though completely processed was found to be catalytically active and therefore could still possibly check downstream cell migration signaling pathways. In fact, both PTPRK-mut and PTPRK-del decreased levels of EGFR and β-catenin which are among major players in cell migration.

Furthermore, our results showed that limiting glioma infiltration and migration by expression of functional PTPRK results in an improved response to several anti-glioma agents. The rationale is that infiltrative behavior of malignant glioma cells into surrounding brain parenchyma that makes current treatments less effective is highly dependent on PTPRK genetic status. Indeed, U87-MG and U251-MG cells ectopically expressing PTPRK-wt showed significantly higher response to temozolomide and/or erlotinib/gefitinib treatment. Thus PTPRK-wt expression significantly increased the drug efficacy. However, non-synonymous mutations or deletions abrogated this observed improvement. This variation in clinical response to chemotherapy could be explained by differences in phosphatase activity, post-translational modifications, and consequent differences in regulation of EGFR and β-catenin signaling. Moreover, one of the major mechanisms of resistance to gefitinib and erlotinib is overexpression of ATP binding cassettes efflux carriers (ABCt). ABCt transporters are activated by phosphorylation [33] and inactivation of certain ABCt by PTPRK might explain improvement in clinical response to these drugs. However, the exact molecular mechanism is as yet unclear and requires further investigation.

Another interesting aspect to note was that although erlotinib and gefitinib are structurally related and have a similar mechanism of action, glioma cells showed better response to treatment with gefitinib than erlotinib, either alone or in combination with temozolomide. This difference in response was enhanced by PTPRK-wt expression. Poor response to erlotinib monotherapy has been previously attributed to PTEN and EGFR mutations (EGFRvIII) in glioma cells [22]. However, response to gefitinib treatment was contrary to recent glioma study results [23]. Another study has recently shown cooperative effect of erlotinib and temozolomide combination therapy at lower doses (1 µM and 25 µM, respectively) in glioma cells [34]. However, this synergistic effect at sub-optimal doses of erlotinib (2.5 µM) and temozolomide (25 µM) was not observed in our experimental conditions (data not shown). We observed only 10–20% decrease in viable cells with combination of erlotinib (2.5 µM) and varying range of temozolomide concentrations (25 µM, 100 µM and 200 µM) relative to untreated U87-MG cells (data not shown). Moreover, the relationship between PTPRK status (cell-cell contact) and temozolomide (targets DNA repair) is currently unclear. A recent study in glioma showed a possible cooperation between EGFR inhibition and temozolomide related reduction in tumor growth [34]. Based on this study, it can be speculated that PTPRK-mediated check on EGFR signaling improves response of PTPRK expressing cells to temozolomide treatment. Overall, our results clearly indicate that PTPRK expression in glioma cells improves clinical response to these anti-cancer drugs, *in vitro*.

## Conclusions

In summary, our findings provide compelling evidence in support of tumor-suppressive properties of PTPRK and its prognostic significance in glioma along with discovery of several mutations leading to altered functionality of the PTPRK protein which subsequently affect therapeutics. The results demonstrate biological relevance of PTPRK loss in glioma pathogenesis. However, it is as yet unclear if the maintenance of required PTPRK expression levels could serve as a sufficient “checkpoint” in the molecular events leading to glioma development and progression. Future studies require further analysis of PTPRK variants in a larger group of glioma patients. Mutational screening in an alternative glioma population will validate the significance of identified mutations. Although PTPRK interacts with β-catenin and EGFR, *in vitro*, it is likely that the cellular substrates of its phosphatase activity vary depending upon subcellular localization of PTPRK fragments following post-translational processing. In future, with data herein being confirmed using *in vitro* and *in vivo* models, PTPRK may be used as a therapeutic predictive marker for tyrosine kinase inhibitors and other anti-glioma agents.

## Supporting Information

Figure S1 **PTPRK Sequences alignment.** Sequence traces depicting the five mutations of PTPRK in coding regions of PTPRK transcripts from glioma patient samples. Sequences alignment was performed using DNAbaser program. The reference sequence NM_001135648.1 was used as a template.
(TIF)

Figure S2 **Post-translational processing of PTPRK in malignant glioma.** (A) A diagrammatic representation of post-translational modification of PTPRK in glioma cells. PTPRK is processed in a sequential manner by activity of three proteases 1) furin-like protease yielding PTPRK E- and P-subunit fragment, 2) α-secretase yielding a PΔE subunit, and 3) γ-secretase that generates a membrane-free PTPRK-intracellular domain (ICD) fragment. The phosphatase domain dephosphorylates β-catenin and EGFR and the ICD may possess transcription regulation activity. (B) Post-translational processing of PTPRK protein was analyzed in several glioma tumor specimens by immunoblotting. Positive controls for the full length PTPRK (Ctrl. A: Full length PTPRK) and the PTPRK-ICD fragment (Ctrl. B: GST-fusion ICD transformed bacteria) were used. Full length PTPRK and its fragments are shown: 1) Full length PTPRK, 2) predicted PTPRK P-subunit, 3) predicted PΔE subunit and 4) predicted ICD domain.
(TIF)

Figure S3 **Mutations altered PTPRK growth inhibitory and anti-migratory effects in U87-MG cells.** (A) Cell images were taken 72 h post-transfection to observe growth characteristics of U87-MG cells. (B) Confluent monolayers U87 cells transfected with empty, wild-type or mutants PTPRK were scratched and imaged at 0 and 24 h. Changes in wound area per time were evaluated using TScratch software. Dotted white lines indicate approximate wound edge at 0 h. Scale bar = 200 µm. (C) Images showing invasive behavior of U87 glioma cells transfected with PTPRK clones. Invaded cells were stained with crystal violet, photographed under an inverted light microscope using 10× and 25× objectives, and quantified by manual counting and using ImageJ software in five randomly selected areas.
(TIF)

Figure S4 **Mutations altered suppressive effects of PTPRK on growth and migration of U251-MG cells.** (A) Images of transfected cells were taken after 72 h to observe growth characteristics of U251-MG cells. (B) Confluent monolayers U251 cells transfected with empty, wild-type or mutants PTPRK were scratched and imaged at 0 and 24 h. Changes in wound area per time were evaluated using TScratch software. Approximate position of the wound edge at 0 h is indicated by dotted white lines. Scale bar, 200 µm (C) Images showing invaded U251 glioma cells transfected with PTPRK clones. Photograph of invaded cells was taken with 10× and 25× objective after staining with crystal violet. Numbers of invaded cells were counted manually and using ImageJ software in five randomly selected areas.
(TIF)

Figure S5 **Wild-type PTPRK expression increases sensitivity of glioma cells to chemotherapy.** Association of PTPRK genetic variants with response to therapeutic agents is shown for (A and B) U87-MG cells and (C and D) U251-MG cells. The graphs are plotted after controlling for anti-growth effect of PTPRK expression.
(TIF)
