# Supplementary figures and images for: Tumor Derived Mutations of Protein Tyrosine Phosphatase Receptor Type K Affect Its Function and Alter Sensitivity to Chemotherapeutics in Glioma
Source: PLoS One. 2013 May 16;8(5):e62852. doi: 10.1371/journal.pone.0062852 (PMC3656086; doi:10.1371/journal.pone.0062852)

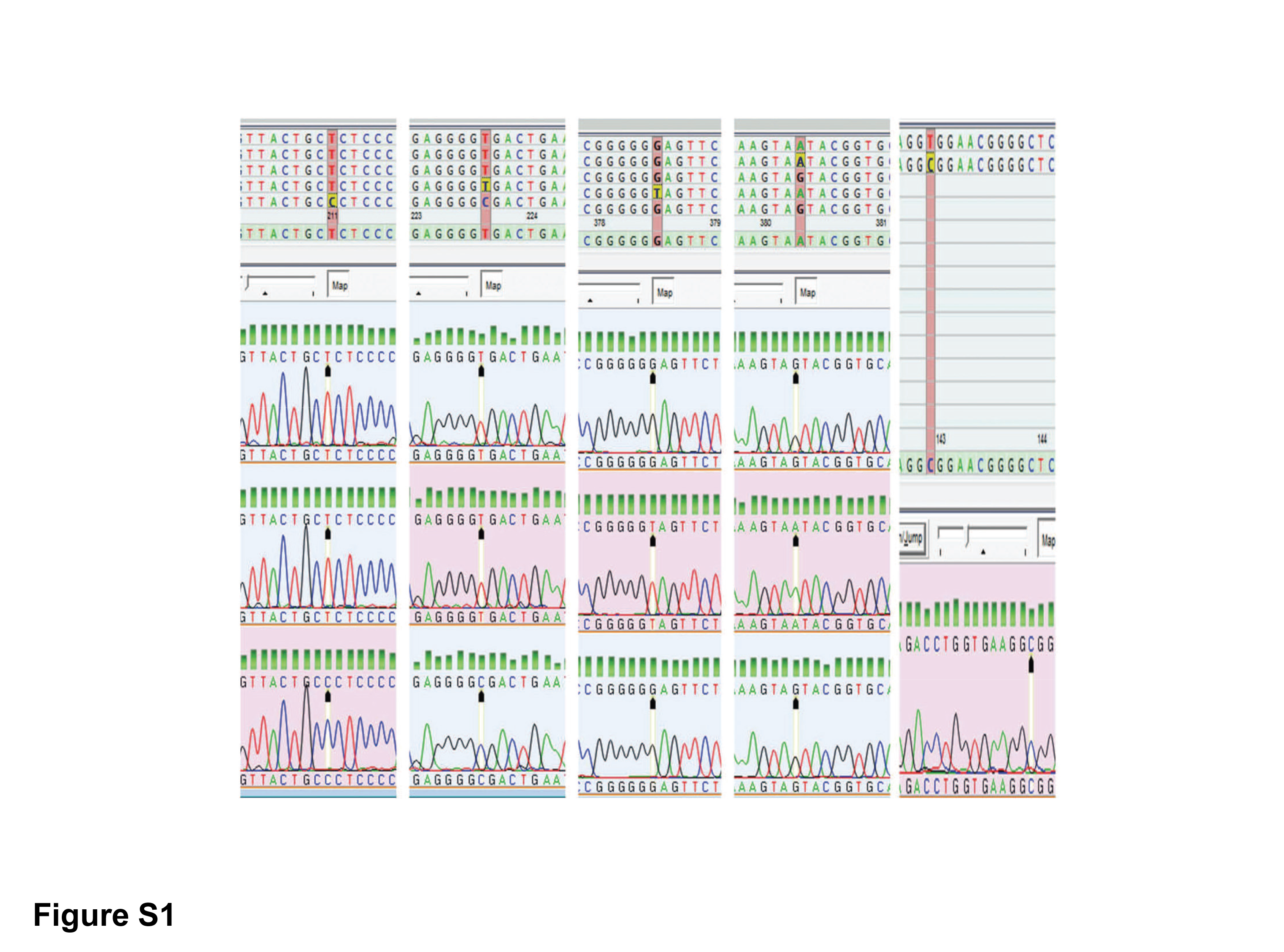

Supplement: Figure S1 — PTPRK Sequences alignment. Sequence traces depicting the five mutations of PTPRK in coding regions of PTPRK transcripts from glioma patient samples. Sequences alignment was performed using DNAbaser program. The reference sequence NM_001135648.1 was used as a template. (TIF) [file pone.0062852.s001.tif]

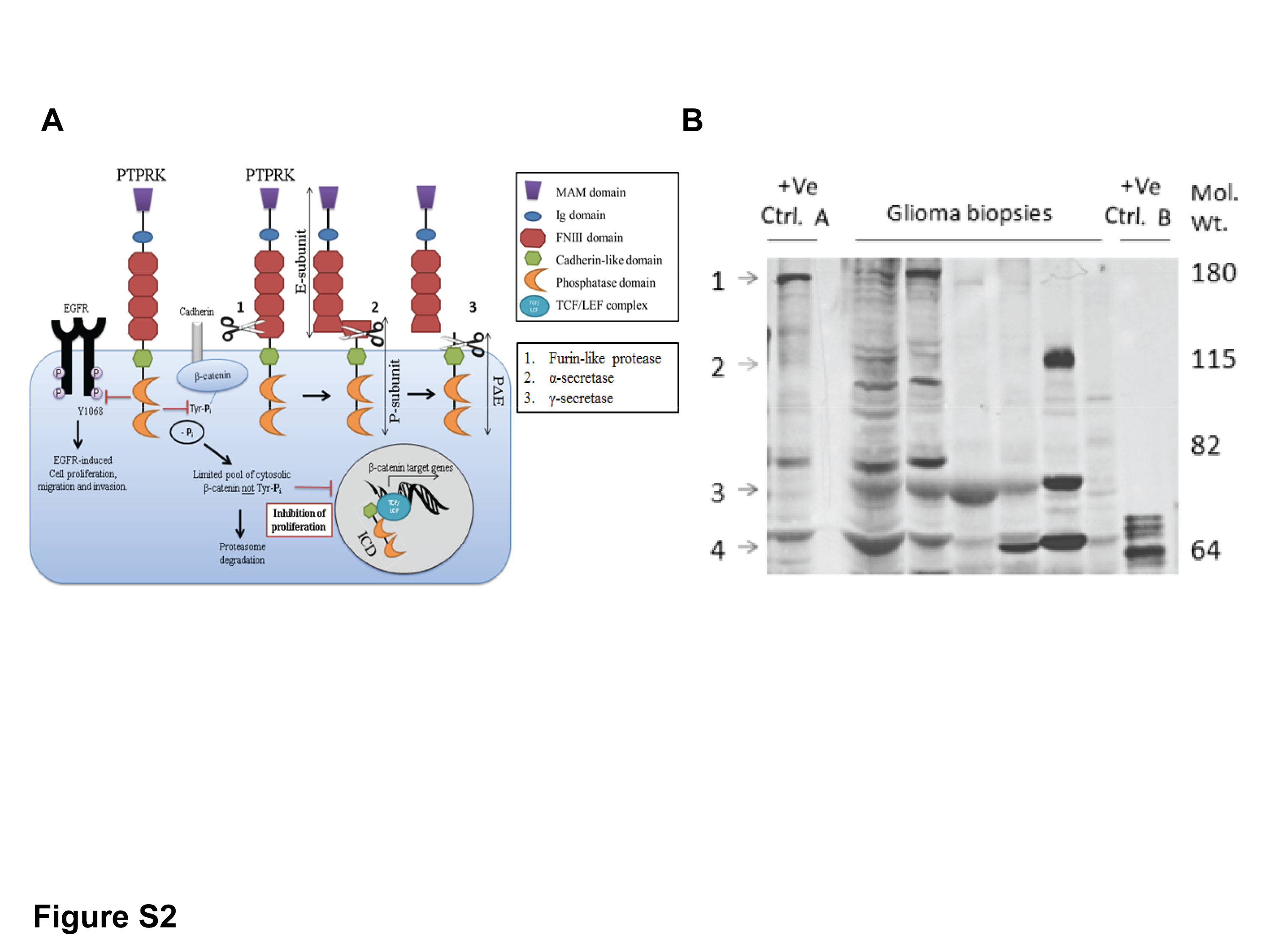

Supplement: Figure S2 — Post-translational processing of PTPRK in malignant glioma. (A) A diagrammatic representation of post-translational modification of PTPRK in glioma cells. PTPRK is processed in a sequential manner by activity of three proteases 1) furin-like protease yielding PTPRK E- and P-subunit fragment, 2) α-secretase yielding a PΔE subunit, and 3) γ-secretase that generates a membrane-free PTPRK-intracellular domain (ICD) fragment. The phosphatase domain dephosphorylates β-catenin and EGFR and the ICD may possess transcription regulation activity. (B) Post-translational processing of PTPRK protein was analyzed in several glioma tumor specimens by immunoblotting. Positive controls for the full length PTPRK (Ctrl. A: Full length PTPRK) and the PTPRK-ICD fragment (Ctrl. B: GST-fusion ICD transformed bacteria) were used. Full length PTPRK and its fragments are shown: 1) Full length PTPRK, 2) predicted PTPRK P-subunit, 3) predicted PΔE subunit and 4) predicted ICD domain. (TIF) [file pone.0062852.s002.tif]

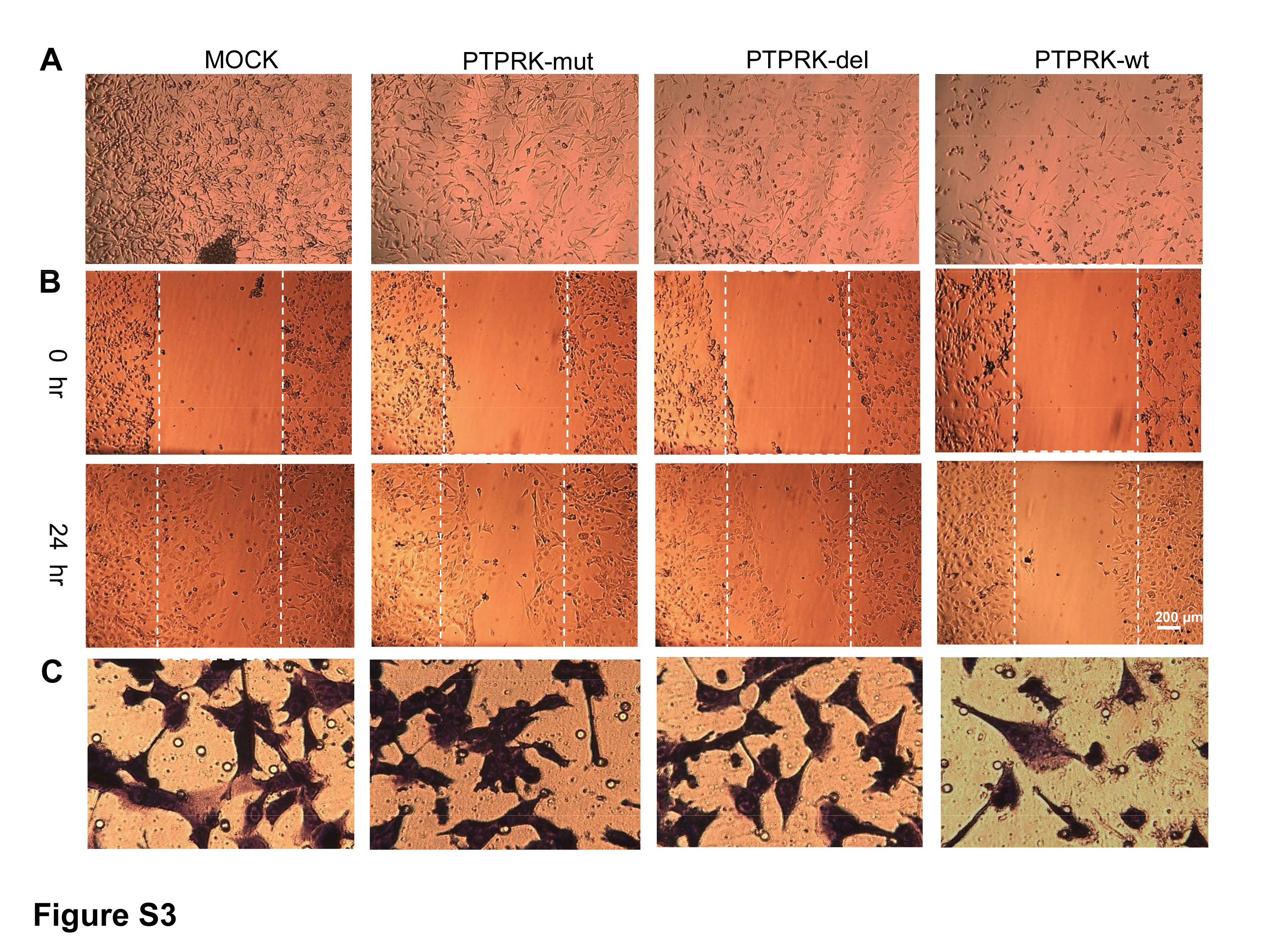

Supplement: Figure S3 — Mutations altered PTPRK growth inhibitory and anti-migratory effects in U87-MG cells. (A) Cell images were taken 72 h post-transfection to observe growth characteristics of U87-MG cells. (B) Confluent monolayers U87 cells transfected with empty, wild-type or mutants PTPRK were scratched and imaged at 0 and 24 h. Changes in wound area per time were evaluated using TScratch software. Dotted white lines indicate approximate wound edge at 0 h. Scale bar = 200 µm. (C) Images showing invasive behavior of U87 glioma cells transfected with PTPRK clones. Invaded cells were stained with crystal violet, photographed under an inverted light microscope using 10× and 25× objectives, and quantified by manual counting and using ImageJ software in five randomly selected areas. (TIF) [file pone.0062852.s003.tif]

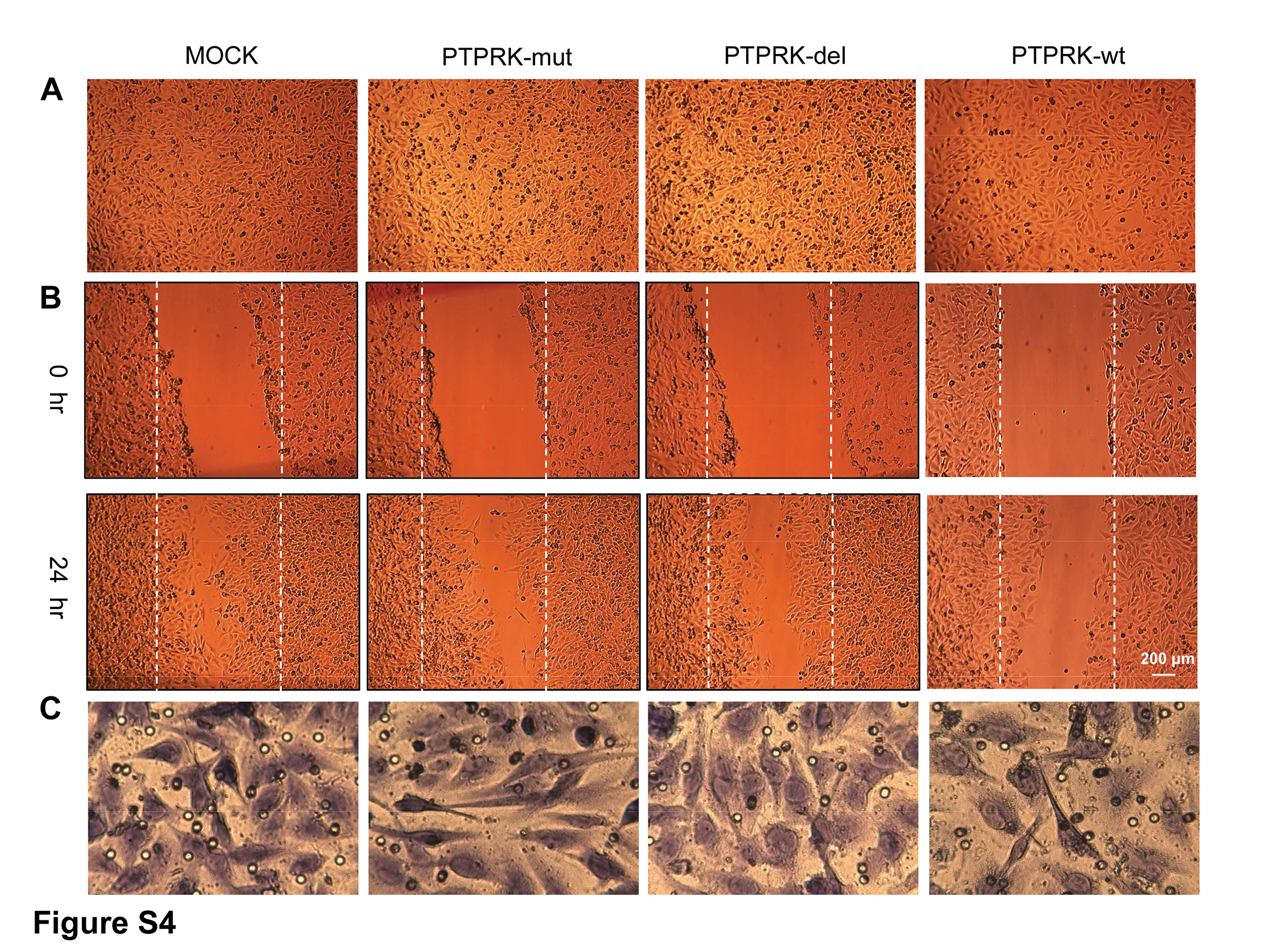

Supplement: Figure S4 — Mutations altered suppressive effects of PTPRK on growth and migration of U251-MG cells. (A) Images of transfected cells were taken after 72 h to observe growth characteristics of U251-MG cells. (B) Confluent monolayers U251 cells transfected with empty, wild-type or mutants PTPRK were scratched and imaged at 0 and 24 h. Changes in wound area per time were evaluated using TScratch software. Approximate position of the wound edge at 0 h is indicated by dotted white lines. Scale bar, 200 µm (C) Images showing invaded U251 glioma cells transfected with PTPRK clones. Photograph of invaded cells was taken with 10× and 25× objective after staining with crystal violet. Numbers of invaded cells were counted manually and using ImageJ software in five randomly selected areas. (TIF) [file pone.0062852.s004.tif]

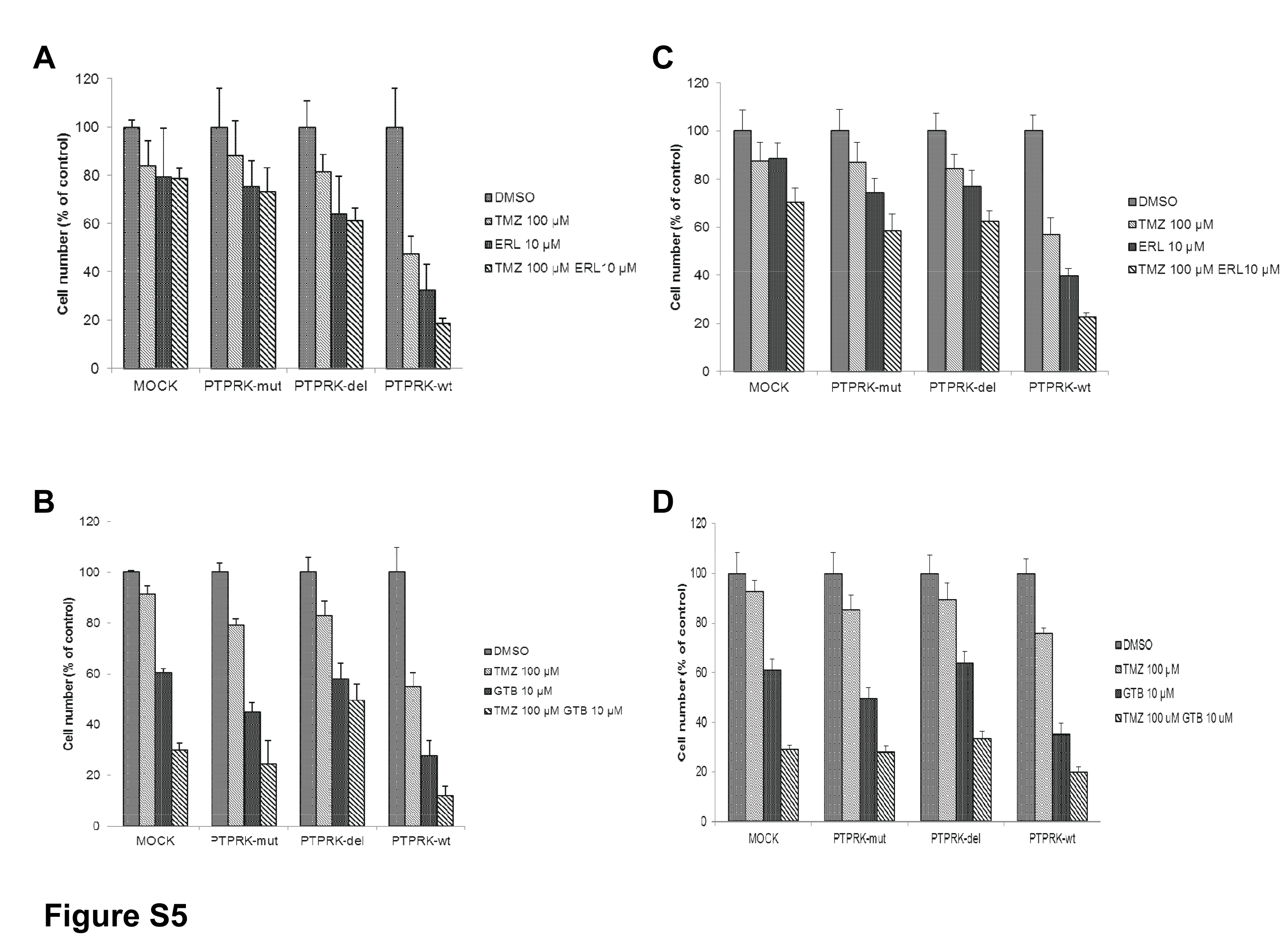

Supplement: Figure S5 — Wild-type PTPRK expression increases sensitivity of glioma cells to chemotherapy. Association of PTPRK genetic variants with response to therapeutic agents is shown for (A and B) U87-MG cells and (C and D) U251-MG cells. The graphs are plotted after controlling for anti-growth effect of PTPRK expression. (TIF) [file pone.0062852.s005.tif]
